# Spatiotemporal characteristics of muscle patterns for ball catching

**DOI:** 10.3389/fncom.2013.00107

**Published:** 2013-08-07

**Authors:** M. D'Andola, B. Cesqui, A. Portone, L. Fernandez, F. Lacquaniti, A. d'Avella

**Affiliations:** ^1^Laboratory of Neuromotor Physiology, Santa Lucia FoundationRome, Italy; ^2^Department of Systems Medicine, University of RomeRome, Italy; ^3^Centre National de la Recherche Scientifique, Aix-Marseille Université, ISM UMR 7287Marseille cedex, France; ^4^Center of Space Biomedicine, University of RomeRome, Italy

**Keywords:** muscle synergies, interception, EMG activity, intermittency, time-to-contact

## Abstract

What sources of information and what control strategies the central nervous system (CNS) uses to perform movements that require accurate sensorimotor coordination, such as catching a flying ball, is still debated. Here we analyzed the EMG waveforms recorded from 16 shoulder and elbow muscles in six subjects during catching of balls projected frontally from a distance of 6 m and arriving at two different heights and with three different flight times (550, 650, 750 ms). We found that a large fraction of the variation in the muscle patterns was captured by two time-varying muscle synergies, coordinated recruitment of groups of muscles with specific activation waveforms, modulated in amplitude and shifted in time according to the ball's arrival height and flight duration. One synergy was recruited with a short and fixed delay from launch time. Remarkably, a second synergy was recruited at a fixed time before impact, suggesting that it is timed according to an accurate time-to-contact estimation. These results suggest that the control of interceptive movements relies on a combination of reactive and predictive processes through the intermittent recruitment of time-varying muscle synergies. Knowledge of the dynamic effect of gravity and drag on the ball may be then implicitly incorporated in a direct mapping of visual information into a small number of synergy recruitment parameters.

## Introduction

Catching or hitting a moving object requires high spatiotemporal accuracy in the control of an end-effector in the presence of visuomotor delays. What sources of information and which visuomotor strategies the central nervous system (CNS) employs to achieve accurate control of interception has been extensively investigated and it is still debated (Zago et al., 2009). On one hand, lawful relationships between visual information available in retinal and extraretinal variables and properties of the target motion, such as time-to-contact or motion in depth, might be exploited to bring the effector on the target (Lee, 1998; Tresilian, 1999; Regan and Gray, 2000). On the other hand, prior knowledge of the characteristics of the target motion and their dependence on environmental conditions, such as gravity acceleration, might be necessary to successfully guide interception (McIntyre et al., 2001; Zago et al., 2004, 2008).

While interceptive movements have been studied extensively in terms of global performance measures and kinematic variables, the underlying muscle activation patterns have received considerably less attention. Even so, the analysis of the timing of muscle activation in preparation for interception has provided evidence for an accurate estimate of time-to-contact. The anticipatory electromyographic (EMG) activity recorded in elbow muscles when catching a ball falling on the hand from different heights shows an early component and a late component (Lacquaniti and Maioli, 1989). The early component has a roughly constant latency from the time of ball release. In contrast, the late component has a constant onset time with respect to the time of impact, indicating that the time-to-contact is estimated accurately. As the ball accelerates under gravity during the fall, precise estimation of time-to-contact cannot be obtained from first order derivatives of retinal variables, as the inverse of the rate of dilation of the retinal image or tau variable (Lee et al., 1983), and requires a-priori knowledge of gravity acceleration (Zago et al., 2008). The onset of EMG activity with respect to the time of impact is also constant for catching of balls thrown frontally (Savelsbergh et al., 1992).

However, to date, the muscle synergies underlying the coordination of multi-muscle EMG activity have not been characterized in catching tasks. It has recently been shown that modulation and superposition of a few time-varying muscle synergies, each appropriately scaled in amplitude and duration and shifted in time, capture the variation of the muscle patterns during reaching to stationary targets in different directions, with different speeds, and after a sudden change of the target location (d'Avella et al., 2006, 2008, 2011b). Such synergies are time-varying as they represent a collection of activation waveforms expressing a balance of muscle activation that varies over a few hundred milliseconds of the time-course of the muscle pattern for a single movement but, as a whole, invariant across movement conditions and repetitions. To test whether an accurate estimation of time-to-contact is used to control the execution of the final phase of an naturalistic and unconstrained interceptive movement and, more generally, to gain insight on the underlying control mechanisms, here we characterized the spatiotemporal organization of muscle patterns during one-handed catching of balls projected from a fixed location and reaching the subject's frontal plane at two different heights (above and below the subject's shoulder height) after three different flight times (550, 650, and 750 ms). This task involves projectile motion as in the previous study by Savelsbergh et al. (1992), but it requires bringing the hand at a point which is not specified in advance, in contrast with the fixed hand position of Savelsbergh et al. (1992). In our conditions, if the effector starts to move well in advance of the latest time at which visual information can affect its trajectory before impact, it is not clear how to detect a signature of an accurate estimate of time-to-contact in the on-going EMG patterns. Here we show that time-varying synergies allow to identify two partially overlapping components in the muscle patterns for intercepting ball with different flight characteristics: one aligned with the ball launch and a second timed to the ball impact.

## Materials and methods

### Subjects, apparatus, and experimental protocol

Six right handed subjects (4 males and 2 females, between 22 and 47 years old) gave their written informed consent to participate in the study, which conformed with the Declaration of Helsinki and had been approved by the Ethical Review Board of the Santa Lucia Foundation. These subjects are a subset of the 14 subjects included in our previous report focusing on task performance and wrist kinematics (Cesqui et al., 2012) for which EMG data was also available. Both the experimental apparatus and protocol have been described in detail previously (Cesqui et al., 2012). Briefly, subjects were asked to catch with the right hand a lightweight (20 g) ball (diameter 7 cm, size similar to that of a cricket or tennis ball) projected in space with different initial velocities by a launching system, built specifically for these experiments (d'Avella et al., 2011a), which allowed to control accurately the mean ball flight time and arrival height at the subject's frontal plane. The ball was projected through a hole in a large screen (4 × 3 m, width × height) at 6 m from the subject's right shoulder and flew along a vertical plane passing through the center of the hole and the shoulder. Subjects were instructed to wait for the ball launch standing with their arms beside their body. Before starting the experiment the system was calibrated to deliver the ball with 3 different flight times (*T*_1_ = 0.55 s, *T*_2_ = 0.65 s, *T*_3_ = 0.75 s) at 2 different heights (*Z*_1_ = 1.3 m, low launches, and *Z*_2_ = 1.9 m, high launches) for a total of six different ball flight conditions. Each subject performed at least 1 block of at least 10 trials for each condition (typically, 3 blocks with 10–15 trials). The order of block execution was selected at random for each subject.

### Data acquisition

The spatial position of the ball (covered with retro-reflective tape, Scotchlite, 3M, Pioltello, Milan, Italy) was measured together with the spatial position of 8 optical retro-reflective markers placed on the subject's trunk and arm close to the following anatomical landmarks: seventh cervical vertebra (C7), clavicle (CL), sternum (SRN), right acromion (RSHO), right epicondylus lateralis (RELB), right forearm (RFRA), right wrist ulnoid styloid (RWRU), right wrist radial styloid (RWRR). Marker positions in space were recorded with a sampling frequency of 100 Hz using a motion capture system (9-camera Vicon-612 system, Vicon, Oxford, UK). A very large tracking volume (6 × 3 × 3 m^3^) was required for capturing the motion of both the ball and the subject upper limb. The marker reconstruction residual, averaged over the nine cameras, obtained in such volume with the Vicon calibration procedure ranged across subjects between 0.93 and 1.03 mm (mean 0.97 mm). Markers coordinates were referred to a right handed calibration frame placed on the floor at 6 m distance from the launch plane, oriented with the *x* axis directed along the antero-posterior axis on the horizontal plane pointing toward the screen and with the *z* axis on the vertical plane pointing upward.

EMG activity was recorded with active bipolar surface electrodes (*DE 2.1*; Delsys, Boston, MA) from the following 16 muscles: biceps brachii, short head (abbreviated as BicShort), biceps brachii, long head (BicLong), brachioradialis (BrRad), triceps brachii, lateral head (TrLat), triceps brachii, long head (TrLong), anterior deltoid (DeltA), middle deltoid (DeltM), posterior deltoid (DeltP), pectoralis major, clavicular portion (PectClav), pectoralis major, sternal portion (PectLow), superior trapezius (TrapSup), middle trapezius (TrapMid), inferior trapezius (TrapInf), latissimum dorsi (LatDors), teres major (TeresMaj), infraspinatus (InfraSp). Each electrode consisted of two parallel silver bars (10 mm spacing) and a differential preamplifier (gain, 10; rms noise, 1.2 μV; common mode rejection ratio, > 80 dB) housed in a compact case (41 × 20 × 5 mm). Electrodes were taped on the muscle belly and connected to an amplifier (Bagnoli-16, Delsys) where the EMG signal was band-pass filtered (20–450 Hz), amplified (total gain 1000) and digitized at 1 KHz by a A/D board in the Vicon system, synchronized with the kinematic data acquisition. For each muscle, correct electrode placement was tested by asking the subject to perform a number of maneuvers involving both free movements and isometric contractions and monitoring the resulting activation patterns on a computer screen.

### Data analysis

All analyses were performed with custom software written in Matlab (Mathworks, Natick, MA). We considered trials in which the subjects either intercepted but did not catch the ball or caught the ball thus excluding trials in which the subjects missed the ball (0.9% of the total number of trials on average). Motion capture and EMG data were analyzed between 200 ms before ball launch and 100 ms after the interception. The time of ball launch was obtained by detecting the instant at which the ball passed through the hole on the screen using a photo-sensor (E3T-S112, Omron Electronics S.p.A., Milan, Italy) mounted on the edge of the hole. The time of interception was obtained by computing the instant at which the distance between the ball trajectory (spatial coordinates as a function of time) and the plane passing through the wrist (RWRU, RWRR) and forearm (RFRA) marker positions reached its minimum (Cesqui et al., 2012). The onset time of wrist movement was defined as the time at which the tangential velocity of the averaged position of RWRU and RWRR markers (marker RWRM) crossed a threshold equal to 10% of its maximum.

#### EMG pre-processing

A notch-filter was used to reduce 50 Hz line noise when present (quality factor 35; Matlab iirnotch function). The EMGs for each trial were digitally full-wave rectified, low pass filtered (20th order FIR filter; 50 Hz cutoff; zero phase distortion; Matlab fir1 and filtfilt functions) and resampled at 200 Hz. Diagnostic screening of EMG signal quality was performed by checking the maximum level of activation, the activation level before the launch time (i.e., with the arm at rest), and the power spectral density of each channel. Eighteen trials (out of a total of 581 trials) that showed high pre-launch activity or abrupt change in signal amplitude, likely resulting from a partial detachment of the electrode from the skin, were excluded. Presence of significant cross-talk was assessed by computing the mean cross-correlation between pairs of channels across trials. Across all subjects, the maximum of the absolute value of the mean cross-correlation was above 0.4 for 11 pairs of muscles (TrLat-TrLong in four subjects, BicShort-BicLong and TeresMaj-InfraSp in three subjects, BicLong-TrLat and PectLow-BrRad in two subjects, TrapInf-TrapMid, DeltA-DeltM, DeltM-TrLat, DeltP-TrLat, PectLow-DeltP, DeltP-BrRad in one subject). Because of the difficulty in distinguishing cross-talk due to volume conduction from synchronous recruitment of motor units in different muscles, we did not remove these muscles from the set used for the main analyses. However, in additional analyses, we verified that the removal of the muscles potentially affected by cross-talk did not change any conclusion drawn from the main analyses. Baseline activity, defined as the mean EMG activity in the 200 ms before launch, was subtracted from each EMG channel. EMGs recorded in each trial were aligned to launch time and averaged across repetitions on each one of the six launch conditions. Finally, the averaged EMG of each channel was normalized to its maximum amplitude across conditions.

#### Time-varying muscle synergies

We modeled the construction of a muscle pattern by combination of N time-varying muscle synergies as follows (d'Avella et al., 2006):

$$m\left(t\right)=\sum_{i\;=\;1}^{N}c_{i}w_{i}\left(t-t_{i}\right)+\epsilon (t)$$

where **m**(t) is a vector of real numbers, each component of which represents the activation of a specific muscle at time *t*; **w**_*i*_(τ) is a vector representing the muscle activation for the *i*-th synergy at time τ after the synergy onset; *t*_*i*_ is the time of synergy onset; *c*_*i*_ is a non-negative scaling coefficient; ϵ(*t*) is the residual. The extraction algorithm was initialized by choosing random values for *N* synergies of a specific duration, and it proceeded by iterating three steps (d'Avella and Bizzi, 2005; d'Avella et al., 2006): (1) synergy onset time estimation, given the synergies; (2) amplitude coefficient estimation, given the synergies and their onset times; (3) synergy update, given onset times and amplitude coefficients.

We used a convergence criterion of five consecutive iterations in which the decrease in the error was 10^−4^. To minimize the probability of finding local minima, for each N we repeated the optimization 20 times and selected the solution with the highest goodness of reconstruction. Because the EMG patterns and the residual of the reconstruction of patterns by synergy combination are multivariate time-series, we used the fraction of total variation explained by the model as a measure of the goodness of the reconstruction (*R*^2^ = 1 – SSE/SST, where SSE is the sum of the squared residuals and SST is the sum of the squared residual for the mean activation of each channel).

**Figure 1:**
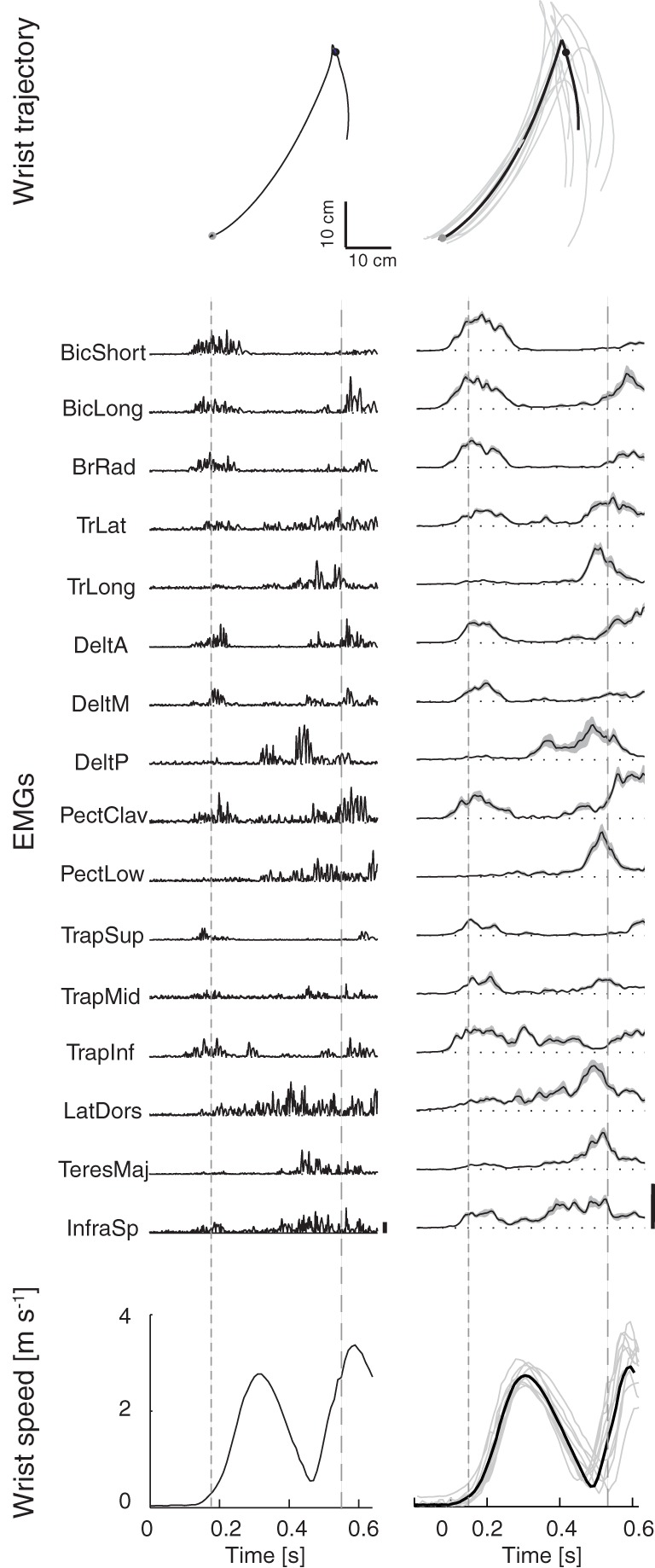
**Example of wrist kinematics and EMGs profiles**. *First column*: example of wrist path in the sagittal plane **(top)**, rectified EMGs **(middle)**, and wrist speed profile **(bottom)** for one trial of subject S_1_ catching a ball with flight duration *T*_1_ and arrival height *Z*_1_. *Second column*: wrist trajectories, filtered EMGs, and wrist speed profiles averaged across multiple trials of subject S_1_ in the condition (*T*_1_,*Z*_1_). *Thick black lines* represent averages across trials, *gray areas* represent the standard deviation; EMGs were aligned to the launch time and normalized to the maximum of each channel across all trials and all launch conditions (*vertical bars*). Times of wrist movement onset and of ball impact with the hand are indicated respectively by the *dashed lines* in the EMGs and speed profiles plots and by *gray circular markers* in the wrist trajectory plots.

**Figure 2:**
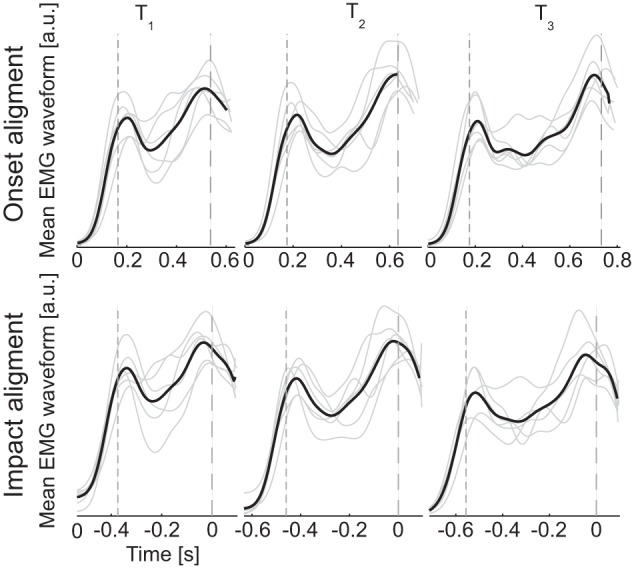
**Gross temporal structure of muscle patterns**. EMG waveforms averaged across muscles and ball arrival height conditions for individual subjects (*gray thin lines*) and averaged across subjects (*black thick lines*). Dashed lines represent respectively the onset (*small dashes*) and impact (*large dashes*) events. Each channel of the EMG averaged waveforms was normalized to its maximum amplitude across all movement conditions before averaging across channels. In the top panels waveforms are aligned to the onset time, in the bottom panel waveforms are aligned to the impact time. Evidence for rough division of the movement in two phases is provided by the presence of two main peaks in all the averaged waveforms.

The number of synergies (*N*) and the duration of each synergy were selected according to the general features of the time-course of the muscle activation waveforms observed (see Figures 1, 2). In particular, we selected 2 synergies with a duration of 400 ms as the minimum number of synergies with the shortest duration which could fully capture the two distinct bursts of EMG activation. We also verified that the reconstruction *R*^2^ did not increase significantly with synergies longer than 400 ms.

Synergies extracted from individual subjects were compared after grouping them with a hierarchical clustering procedure based on distance between synergy pairs. Distance between two synergies was defined as 1 − s, where s is the similarity between two synergies defined as the maximum of the scalar product between the two normalized synergies across all possible relative time shifts (d'Avella et al., 2006). A hierarchical tree was constructed from the distances between all pairs (Matlab function linkage) and such tree was used to form clusters (Matlab function cluster; “cutoff” parameter 0.15).

As the highest similarity between synergies in the same cluster was obtained with a non-zero relative time shift, the value of such time shift with respect to the synergies of subject S_1_ was subtracted from the timing coefficient (*t*_*i*_) to compare them across subjects.

To assess the potential effect of catching performance, we compared the synergies extracted from trials in which the ball was caught with those extracted from trials in which the ball was intercepted but not caught.

To analyze synergy amplitude coefficients (*c*_*i*_), each synergy was normalized to the maximum of the Euclidian norm of synergy vectors (**w**_*i*_(t)) across time samples and the corresponding coefficient scaled by this norm. Moreover, to compare amplitude coefficient across launch conditions, each coefficient was normalized to its maximum value.

#### Statistical analysis

To explain the effect on the synergies recruiting parameters of experimental condition (three ball flight times and two ball arrival heights), the synergy amplitude and timing coefficients were submitted to either a Two-Way ANOVA test (3 arrival times × 2 height conditions) or to a multiple linear regression analysis according to the model:

$$y=\alpha +\beta T+\gamma Z_{d}+\delta (T,Z_{d})+\varepsilon$$

where *T* is the time variable, i.e., the ball flight time, *Z*_*d*_ is a dummy variable for the ball arrival height *Z*, i.e., *Z*_*d*_ = 1 for low launches and *Z*_*d*_ = 2 for high launches. Statistical analyses were performed in the R software environment (R Development Core Team, 2011). The level of significance was set at *p* < 0.05.

## Results

Subjects, starting with the arm at rest and relaxed along their body, were instructed to catch lightweight balls projected from about 6 m from their shoulder and at 1.66 cm height and arriving at their frontal plane at two different heights and with three different flight times. On average, 67.6 ± 11.8% (mean ± SD, *n* = 6, range 84.6 – 55.0%) of launched balls were successfully caught and 30.8 ± 12.0% (41.7 – 13.5%) were intercepted but not caught. Mean onset time of the wrist movement with respect to ball launch over all trials was 171 ± 15 ms (mean ± SD, *n* = 6, range 149 – 193 ms). Mean movement time was 377 ± 20, 459 ± 21, 538 ± 23 ms (mean ± SD, *n* = 6) for low launches (*Z*_1_) at the three different flight times (*T*_1_, *T*_2_, and *T*_3_) and 370 ± 7, 462 ± 15, and 575 ± 23 ms for high launches (*Z*_2_).

### Muscle patterns

The EMG activity waveforms recorded during catching of a ball flying along a trajectory with a low arrival height and the shortest flight time (condition *Z*_1_ *T*_1_, Figure 1, *first column*, a single trial and, *second column*, average over 10 trials for subject S_1_) illustrate the key features of the patterns observed in all conditions and subjects. Muscles were activated mainly in two phases. Elbow flexors (biceps brachii and brachioradialis) and shoulder forward flexors and scapula elevators (anterior and middle fibers of deltoid, upper trapezius) were among the most active muscles in a first phase during which the hand quickly raised from its initial rest position toward the region of interception. Muscles involved in backward flexion, adduction and internal rotation of the humerus (teres major, posterior deltoid, pectoralis major) or in extension and flexion from extended positions of the humerus (latissimus dorsi), together with elbow extensors (triceps brachii) were instead mostly active in a second phase in which the hand impacted the ball.

The general temporal structure of the muscle patterns for catching and their dependence on the flight time can be grasped from the ensemble averages of the EMG waveforms across muscles and trials with the same flight time. Two distinct peaks are clearly visible in the averaged EMG waveforms (Figure 2). The first peak has an approximately constant latency with respect to the time of ball launch, independently of flight duration [203 ± 14 ms, mean ± SD, *n* = 6, for *T*_1_; 216 ± 18 ms for *T*_2_; 212 ± 11 ms for *T*_3_; One-Way ANOVA, main effect of *T*:*F*_(2, 17)_ = 1.25, *p* = 0.31]. Instead the second peak has an approximately constant lead with respect to the time of impact, independently of flight duration [ −28 ± 16 ms for *T*_1_; −5 ± 19 ms for *T*_2_; −41 ± 26 ms for *T*_3_; One-Way ANOVA, main effect of *T*:*F*_(2, 17)_ = 1. 73, *p* = 0.21]. Thus, the general temporal structure suggests that an initial component of the muscle pattern is generated in response to the detection of the ball launch and a second component is precisely timed to the time of interception. This finding confirms previous results obtained for catching a free-falling ball (Lacquaniti and Maioli, 1989), and extends the observation to unconstrained catching of ballistic projectile motion.

### Time-varying synergies

The novel results concern the synergic organization of muscle activity. To characterize the fine spatiotemporal structure of the muscle patterns, we decomposed them as the combination of two time-varying muscle synergies, each one with a condition-specific amplitude and onset time. Each synergy had a duration of 400 ms, spanning the duration of both EMG activation phases in all conditions. The reconstruction *R*^2^ was on average 0.73 ± 0.07 (mean ± SD, *n* = 6, range 0.65 – 0.83) indicating that a decomposition into two time-varying muscle synergies adequately captured the key features of the EMG waveforms.

**Figure 3:**
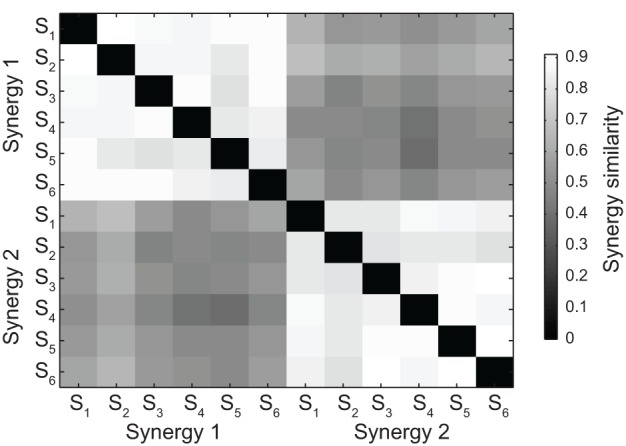
**Synergy grouping by hierarchical clustering based on pair similarity**. The grayscale matrix shows the similarity between all pairs of time-varying synergies extracted from all subjects (two synergies for each subject) defined as the maximum of the scalar product between the two normalized synergies across all possible relative time shifts. Synergy pairs in the matrix are grouped into two clusters using a hierarchical clustering algorithm (see Materials and Methods).

The set of all pairs of synergies extracted from all subjects could be grouped according to their similarity into two clusters (see Materials and Methods), each contacting only one of the two synergies extracted from each subject (Figure 3). The mean similarity between pairs of synergies was 0.86 ± 0.03 (mean ± SD, *n* = 15, range 0.50 – 0.91) in the first cluster and 0.85 ± 0.04 (mean ± SD, *n* = 15, range 0.55 – 0.88) in the second cluster. Moreover, the structure of the synergies was not affected by catching performance. The mean similarity between synergies extracted from all trials and synergies extracted only from trials in which the ball was caught was 0.94 ± 0.04 (mean ± SD, *n* = 6, pairs of synergies in the two conditions for each subject) for the first synergy and 0.92 ± 0.05 for the second synergy.

**Figure 4:**
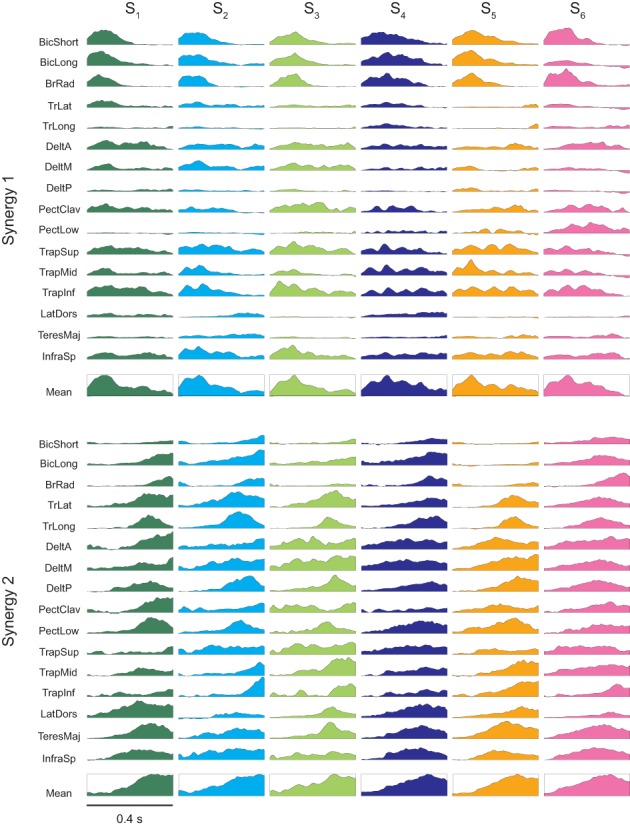
**Time-varying muscle synergies**. Time varying muscle synergies are shown for all subjects (color coded). The first synergy (**top row**) captured mostly the EMG patterns related to the initial phase of the movement in reaction to the ball launch. The second synergy (**bottom row**) captured mostly the EMG patterns related to the final interceptive phase of the movement. Below the set of muscle waveforms constituting each muscle synergy their mean waveform is illustrated within a box.

The synergies in both clusters captured synchronous and asynchronous activations of many muscles. The synergies of the first cluster (Figure 4, *first row*) recruited strongly elbow flexors (biceps brachii and brachioradialis), shoulder flexors (anterior deltoid and the clavicular portion of pectoralis) and shoulder elevators (superior trapezius) with a shorter bursts in the elbow flexors than in the other muscles in most cases. The synergies of the second cluster (Figure 4, *second row*) showed a higher level of co-activation of the entire set of muscles but also a more complex temporal structure and inter-individual variability than the synergies of the first cluster. Elbow extensors (triceps brachii, long and lateral heads), shoulder extensors and adductors (posterior deltoid, lower portion of pectoralis, latissumus dorsi) were strongly recruited with a burst peaking before the end of the synergy, while other muscles (biceps brachii long head, anterior deltoid) were recruited later and showed a ramped activation.

### Synergy recruitment modulation across conditions

**Figure 5:**
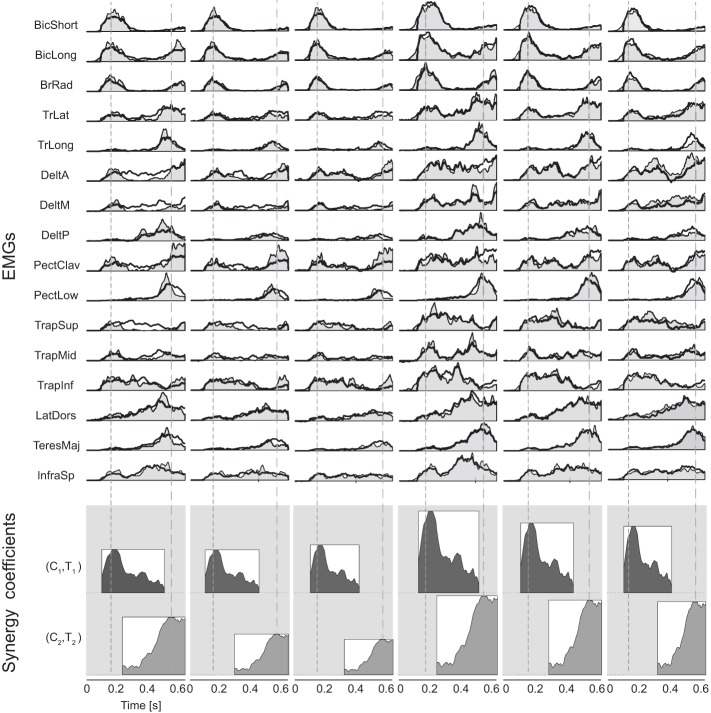
**Example of reconstruction of muscle patterns by synergy combination**. Recorded muscle activation waveforms (*light gray area*) in all movement conditions for subject S_1_ are reconstructed (*thick black line*) by the combination of two synergies (amplitude and timing coefficients represented by the height and the horizontal position of the rectangles with the mean activation waveform at the bottom of each panel). Onset and interception time are represented respectively by the dashed lines. The patterns are aligned to the launch time and normalized to the maximum amplitude for each EMG channel across all the six conditions. For all launch conditions, the first synergy was aligned to the launch time and the second synergy was aligned to the impact time.

The averaged EMG patterns of subject S_1_ for all the launch conditions (Figure 5, top, gray shaded area) and their reconstruction as combination of the two synergies (black line) illustrate how the synergy amplitude and timing coefficients (represented respectively by the height and the horizontal position of the rectangles below the EMG waveforms) were modulated across conditions. In all six conditions, the first synergy was aligned with the launch time (first dashed vertical line of Figure 5). Across all subjects, a Two-Way ANOVA (3 flight times × 2 arrival heights conditions) showed that the ball flight time and arrival height did not affect the latency time of the first synergy with respect to the ball launch [main effect of *T*:*F*_(2, 35)_ = 2.24, *p* = 0.124, main effect of *Z*:*F*_(1, 35)_ = 1.41, *p* = 0.244, interaction:*F*_(2, 35)_ = 0.22, *p* = 0.801]. Instead, the onset of the second synergy appeared to be aligned with the impact time (second dashed vertical line of Figure 5). Across all subjects, there was a significant effect of the ball flight time but no effect of the ball arrival height [main effect of *T*:*F*_(2, 35)_ = 3.83, *p* = 0.033; main effect *Z*:*F*_(1, 35)_ = 1.92, *p* = 0.156; interaction:*F*_(2, 35)_ = 1.91, *p* = 0.165] on the onset time of the second synergy with respect of impact time. However the observed effect of the ball flight time could be ascribed to subject S_3_ and S_4_, who recruited the second synergy closer to the impact event in the *T*_3_Z_2_ condition (S_3_ and S_4_) and in the *T*_2_Z_3_ condition (S_4_). When we excluded those two subjects, we found no statistically significant effect of either flight time or arrival height [Two-Way ANOVA without S_3_ and S_4_, main effect *T*:*F*_(2, 23)_ = 2.14, *p* = 0.14; main effect *Z*:*F*_(1, 23)_ = 0.03, *p* = 0.85; interaction:*F*_(2, 23)_ = 1.54, *p* = 0.24].

**Figure 6:**
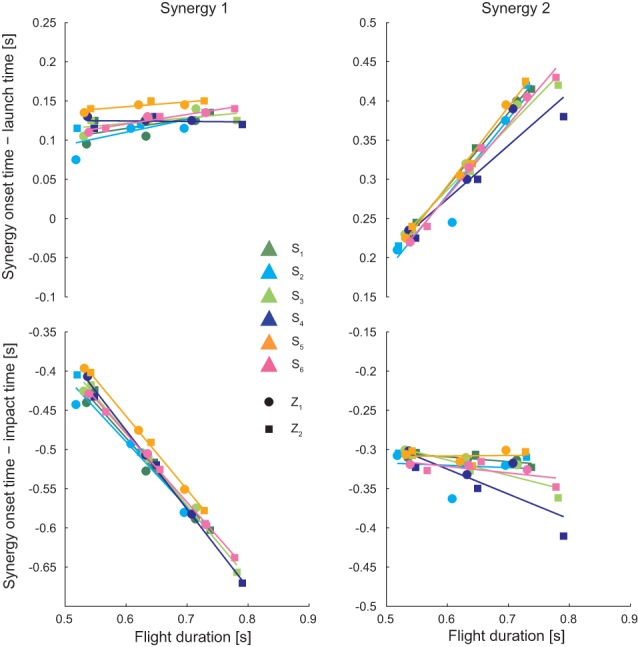
**Synergy onset as a function to the flight time**. The synergy timing coefficients t_i_ are shown with respect to the flight time after time alignment of synergies to the launch time (**top**) and to the impact time (**bottom**), for all subject and all launch conditions (circular markers are referred to low launches, square markers are referred to high launches). Results of linear regression performed subject by subject are also shown. For all subject the onset of the first synergy was constant with respect to the launch time and the onset of the second synergy was constant with respect to the impact time, independently of the flight time.

We also analyzed the onset of the two synergies as a function of ball flight duration (Figure 6) after alignment to the launch time (*top*) and to the impact time (*bottom*) for all subjects (*different colors*) and both low (*circular markers*) and high (*square markers*) launches, as the flight time also depended on the choice of interception point, which varied across conditions and individual. When aligned to launch time, the onset of the first synergy did not depend on ball flight conditions (multiple regression analysis: *p*_β_ = 0.14, *p*_γ_ = 0.24, *p*_δ_ = 0.29), while the onset of the second synergy increased linearly with the flight time (multiple regression analysis; *p*_β_< 0.01 with β = 1.08, *p*_γ_ = 0.149, *p*_δ_ = 0.12). On the contrary, when aligned to impact time, the onset of the first synergy decreased linearly with flight time (multiple regression analysis:*p*_β_< 0.01 with β = − 0.79, *p*_γ_ = 0.25, *p*_δ_ = 0.29) while the timing of the second synergy did not depend on flight time (multiple regression analysis:*p*_β_ = 0.55, *p*_γ_ = 0.149, *p*_δ_ = 0.12).

**Figure 7:**
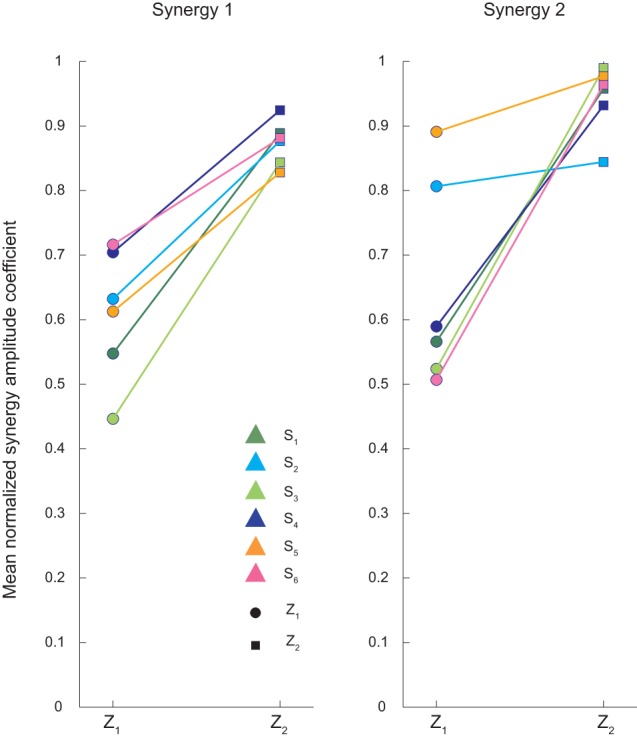
**Averaged synergy amplitude coefficients for the two different ball arrival heights**. Synergy amplitude coefficients, averaged across flight times for low (*Z*_1_) and high (*Z*_2_) launches, are showed for all subjects. The amplitude coefficients of the first synergy increased similarly with ball arrival height in all subjects. The amplitude coefficients of the second synergy showed instead a weaker modulation with arrival height for subject S_2_ and S_5_ than the other four subjects.

The amplitude coefficients *c*_*i*_ of the synergies were also modulated with respect to the ball arrival height. Figure 7 shows the coefficients of individual subjects for each arrival height, normalized to the maximum across all conditions and averaged across flight times. The first synergy coefficient increased with arrival height (multiple regression analysis: *p*_β_ = 0.44, *p*_γ_ = 0.004 with g = 0.75, π_δ_ = 0.0571). In contrast, the amplitude coefficient for the second synergy was modulated differently with arrival height [multiple regression analysis *p*_β_ = 0.02, *p*_γ_ = 0.19, *p*_δ_ = 0.034; Two-Way ANOVA test main effect *T*:*F*_(2, 35)_ = 0.75, *p* = 0.482; main effect *Z*:*F*_(1, 35)_ = 40.93, *p* < 0.01; interaction:*F*_(2, 35)_ = 1.53, *p* = 0.23]. For subjects S_1_, S_3_, S_4_, and S_5_ the coefficient almost doubled going from *Z*_1_ to *Z*_2_, while for subjects S_2_ and S_5_ the coefficient showed only a small increase.

## Discussion

The synergy decomposition of the muscle activity patterns recorded during one-handed catching of fast balls, projected along spatial trajectories with different flight durations and arrival heights, revealed remarkable spatiotemporal characteristics. A large fraction of the variation of the muscle patterns for catching of balls with different flight parameters was captured by amplitude modulation, onset-time shift, and superposition of two time-varying muscle synergies, each representing the coordinated recruitment of a group of muscles with specific temporal activation profiles. The first synergy was recruited with a fixed latency from the time of ball launch and the second synergy with a fixed anticipation of the time of hand-ball impact. These results indicate that the control of the fast hand movements required to intercept a ball with flight duration below 750 ms relies on a combination of reactive and predictive processes. The initial muscular response, captured by the first synergy, was generated as fast as possible after the detection of the ball launch and allowed the subject's hand to reach the interception zone, always above the position of the hand at the time of launch. The following component of the muscle pattern, captured by the second synergy, guided the hand to the interception point in a fixed time interval, i.e., according to an accurate estimation of the time necessary to reach the interception point.

The fact that we could reconstruct the muscle patterns for catching balls with three different flight times and two different arrival heights by combining two time-varying muscle synergies does not imply that the two identified synergies are basic building blocks sufficient to control all possible interceptive movements. Many recent studies of the muscle patterns for the control of the arm have identified muscle synergies by systematically varying the spatial constraints of the task (Muceli et al., 2010; Cheung et al., 2012; Roh et al., 2012; Delis et al., 2013). In particular, in our previous studies of time-varying muscle synergies for reaching (d'Avella et al., 2006, 2008, 2011b) we systematically varied the direction of the movement. In contrast, in this study we have focused on the effect of changing the temporal constraints inherent in an interception task (flight duration) with only two related spatial constraints (arrival height). A much larger set of flight conditions with a variety of arrival locations should be tested to make the synergies identified by a decomposition algorithm from the muscle patterns representative of basic building blocks underlying the generation all possible catching movements. While we plan to test a more diverse set of experimental conditions in the future, the synergy decomposition approach was used here as a tool to identify spatiotemporal components in the muscle patterns independent of temporal constraints, such as flight duration. Thus, the synergies identified from this dataset might be composed by the combination of a larger number of more fundamental building blocks modulated by the spatial demands of the task, such as ball arrival locations at different medio-lateral coordinates.

Our observation of the modulation of the onset time of the second synergy by the temporal demands of the task, i.e., the duration of the ball flight and of the ball arrival at the interception point, may be contrasted with the timing modulation of the synergies for reaching a stationary target. In our previous investigation of the time-varying synergies for reaching with different movement directions and speeds (d'Avella et al., 2008) we found that a set of phasic and tonic synergies captured the muscle patterns once time-normalized to equal movement duration and that the onset of the synergies most active in each movement direction, expressed as a fraction of movement time, was approximately constant over a broad range of movement speeds. Thus, when reaching a stationary target with a movement which could be planned in advance and whose duration did not depend on an online estimation of the ball arrival time, as with an interceptive movement, the entire muscle pattern was scaled with the movement duration and the onset time of the synergies was then shifted accordingly with respect of the movement end. To directly compare the synergy timing for catching and reaching in similar conditions, we considered a subset of the data we had previously collected (d'Avella et al., 2008), selecting only the trials for reaching upward, and we averaged the muscle patterns over the trials with a movement time within three intervals around the mean movement times of the catching movements for the three ball flight time conditions of this study. We then extracted two time-varying muscle synergies from the averaged reaching muscle patterns, aligned to movement onset and time-normalized to equal movement time, as in the original analysis, and with the baseline activity before movement onset subtracted, as in the present analysis. The fraction of data variation explained by two synergies was higher for reaching than for the catching patterns (*R*^2^ = 0.83 ± 0.10, mean ± SD, *n* = 5, range 0.71 – 0.92) and the onset of the second synergy was not aligned with movement end but it occurred closer to the movement end for faster movements than for slower ones, as expected for muscle patterns scaled in time with movement speed. Thus, the modulation of the recruitment timing of the time-varying muscle synergies indicated that the muscle patterns for catching movements with different duration, differently from those for reaching, were not-scaled in time as a unit but consisted of two distinct temporal components, one aligned to the ball launch and movement onset and a second aligned to the time of ball interception and thus shifted progressively later for longer movement times.

The observation of a fixed temporal relationship between muscle synergies and specific task events, ball launch and ball impact with the hand, is in accordance and extends previous observations made in the context of catching balls falling vertically on the hand (Lacquaniti and Maioli, 1989) and of balls launched frontally onto the hand (Savelsbergh et al., 1992). A fixed relationship was found between the onset of an early component of wrist and elbow muscle activity and the time of ball release and between the onset of a late component and the time of ball impact for when catching balls falling from different heights (Lacquaniti and Maioli, 1989). Moreover, the onset of wrist and finger muscle activity showed a fixed relationship with the time of impact for balls launched frontally with different speeds (Savelsbergh et al., 1992). However, in contrast to those studies, in our catching task the location of the interception was not specified prior to ball launch, as the ball could be intercepted anywhere along the portion of the ball trajectory inside the hand workspace, and subjects had to determine not only the appropriate time of interception but also the appropriate place. Thus, in our unconstrained and naturalistic task subjects had to move their hand to the interception location and simultaneously time the closing of the fingers on the ball. Moreover, as all trials started with the arm relaxed along the body and all ball trajectories crossed the hand workspace above that hand initial location, subjects initiated their movement as soon as they detected the ball launch to bring the hand close to the interception region. Indeed, such reactive component was characterized by a time-varying synergy, recruiting mainly elbow flexors, shoulder flexors, and shoulder elevators. The amplitude coefficient of this synergy was modulated by ball flight arrival height (Figure 7), which was known by the subject after the first trial of each block. When catching balls falling vertically on the hand, a conditions in which is it not necessary to displace the hand, the early anticipatory response in elbow (but not in wrist) muscles, is time-locked to the ball release and modulated in amplitude by release height and it has been interpreted as an alertness reaction (Lacquaniti and Maioli, 1989). More generally, the initial component of the interceptive muscle pattern, captured by the first synergy, may be interpreted as the reactive release of a motor program that brings the hand in an adequate spatial location from which a second motor program can be released once the interception location and time has been determined. As the time-to-contact during the ball flight depends on the final interception position and since the onset of the second synergy occurred at about the same time-to-contact, we can speculate that during the first part of the flight the recruitment parameters, activation amplitude and onset time, of the second synergy are selected to intercept the ball at a specific location and time along its trajectory. The selection of the appropriate synergy recruitment parameters might derive from a combination of visual information and a-priori knowledge of the dynamic behavior of the ball under gravity and viscous drag forces (Zago et al., 2009). Thus, in such synergy-based control scheme, a-priory knowledge of gravity and drag on the ball may be implicitly incorporated in a direct mapping of visual information into a small number of synergy recruitment parameters. Non-invariant parameters involved in this mapping, such as those relative to drag, may be learned quickly from experience if such mapping is intrinsically low-dimensional, as it would be if also the dimensionality of visual input is reduced by the extraction of a few spatiotemporal features.

Whether the control of interceptive movement relies on predictive or prospective control is debated (Bootsma and van Wieringen, 1990; Brenner et al., 1998; Dessing et al., 2002; Tresilian, 2005; Zago et al., 2009). According to prospective control strategies, appropriate processing of the visual information directly drives the hand toward the target, thus continuously updating the motor response according to an extrapolation of the trajectory based on sensory feedback rather than on an explicit representation of the trajectory or a prediction of the interception point and timing (Chapman, 1968; McLeod and Dlenes, 1993; Peper et al., 1994; McBeath et al., 1995; Brenner et al., 1998; Montagne et al., 1999; Dessing et al., 2002). In contrast, according to the simplest form of predictive control strategy, a stereotyped motor response of a fixed duration is preprogramed and triggered when the predicted time-to-contact is equal to the sum of the movement duration and the sensorimotor delay (Tyldesley and Whiting, 1975). However, as movement duration and velocity have been observed to depend on the parameters of the interceptive task (Bootsma and van Wieringen, 1990; Smeets and Brenner, 1995; Tresilian and Plooy, 2006), according to a more flexible predictive control strategy movement time and the criterion for triggering the response may be adjusted (Tresilian, 2005). In general, hybrid control strategies combining predictive and prospective control, with different weights depending on the time available for the interceptive movement, may be adopted by the CNS (Tresilian, 2005; Zago et al., 2009; Katsumata and Russell, 2012). For rapid interceptive actions, as those required in our task, the effectiveness of continuous on-line guidance by visual feedback is limited because of sensorimotor delays and subjects might adopt an intermittent control strategy (Burdet and Milner, 1998; Gawthrop et al., 2011; Loram et al., 2011; Karniel, 2013) based on an overlapping sequence of time-varying muscle synergies (d'Avella et al., 2011b). Each synergy might be triggered at a critical time and modulated according to a combination of visual information and a-priori knowledge. This control scheme is related to the biphasic preprogrammed model proposed to explain biphasic movements observed in a one-dimensional hitting task (Tresilian, 2005). In such model a first slow component is initiated at one critical value of time-to-contact and a second faster component at a smaller value. In our three-dimensional interception task, however, the location of the interception is not fixed and known prior to ball launch and the parameters of the interceptive movement cannot be fully pre-programmed. Thus, rather than a process that simply triggers a command when a visually driven time-to-contact signal reaches a critical value dependent on the duration of the preprogrammed movement component, we envision a process that triggers a muscle synergy at a critical time-to-contact value and simultaneously adjusts the synergy amplitude parameters to ensure that the hands is at the right location after that time interval.

**Figure 8:**
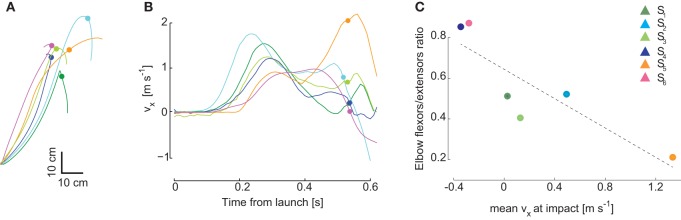
**Inter-individual variability in wrist kinematics and in the structure of the second synergy**. Individual characteristics in the movement kinematics were observed in the several features such as the wrist paths in the sagittal plane, illustrated in **(A)** for condition (*T*_1_,*Z*_1_), and the horizontal component of the wrist velocity in the sagittal plane (v_*x*_), illustrated in **(B)** for the same condition. Subject color coding as in preceding figures; *circular markers* indicate the time of impact. **(C)** Differences across subjects in the mean activation level of elbow extensors and elbow flexors of the second synergy waveforms are related to differences in the mean horizontal wrist velocity at impact across conditions. Subjects S_4_ and S_6_ who intercepted the ball with on average negative (backward) velocity showed a high ratio close to 1 between elbow flexors activation and elbow extensors activation areas. For the subjects who impacted the ball with low velocity (S_1_, S_2_, S_3_) the ratio was near 0.4/0.5, while for subject S_5_, who impacted the ball with a large forward velocity, the ratio was near zero.

In our previous study of the kinematic characteristics of the interceptive movements in this naturalistic, unconstrained catching task we found that individuals with similar performance level used different movement strategies (Cesqui et al., 2012). Also for the six subjects included in this study, inter-individual differences were particularly clear in terms of wrist trajectory in the sagittal plane and wrist velocity at impact (Figures 8A,B). As the second synergy captures mostly the second phase of the movement, we expected differences in the relative recruitment of elbow flexors and extensors. Indeed, it is apparent in Figure 4 that in the second synergy of subjects S_4_ and S_6_, who impacted the ball with on average backward velocity (negative *x* component), both elbow flexors and elbow extensors are highly recruited. In contrast, in the second synergy of subjects S_1_, S_2_ and S_3_, who showed hook-like trajectories and impacted the ball with low horizontal velocity, the elbow extensors are more strongly recruited than the elbow flexors. Finally, in the second synergy of subject S_5_, who impacted the ball with a forward directed movement, the elbow flexors were almost completely silent and the elbow extensors were highly recruited. We then quantified these observations by computing, for the second synergy, the ratio between the mean area of the activation waveforms of the elbow flexor muscles (BicLong, BicShort, BrRad) and the mean of the area of the activation waveforms of elbow extensor muscles (TrLat, TrLong) as a function of the horizontal component on the sagittal plane of the wrist velocity (Figure 8C). The linear regression between the elbow flexors/extensors ratio and the wrist tangential velocity at impact was significant (*R*^2^ = 0.76, *p* = 0.02). Thus, some of the individual characteristics of catching kinematics are reflected in the structure of the synergies. However, despite these inter-individual differences in the structure of the second synergy, the overall spatiotemporal organization of both synergies was remarkably similar across individuals (Figure 3). Additional inter-individual characteristics in the synergy organization can be found when more than two synergies are extracted in each subject. We characterized the muscle patterns with two time-varying synergies because their gross temporal organization in all subject was biphasic (Figure 2) and because two synergies captured always at least 65% of the data variation. However, while in two subjects (S_1_ and S_5_) the *R*^2^ value reached a plateau at 2 synergies [assessed by the detection of a “knee” in the R2 curve, see d'Avella et al. (2006)], in the remaining four subjects (S_2_, S_3_, S_4_, S_6_) the plateau was at three synergies. In these sets of three synergies, one synergy was always similar to the first synergy of the set of two and it showed the same temporal relationship with ball launch. The other two synergies were related to the second synergy in the set of two but they showed additional subject-specific features. In general, one of the two remaining synergies was aligned to the impact time. The other synergy was recruited mainly for high launches and it was timed differently across subjects, generally in between the other two synergies, but with variable onset alignment. Thus, subtle differences in the muscle patterns captured by sets with more than two synergies might contribute to additional individual kinematic features. However, the similarity of two synergies across subjects suggests that individual kinematic strategies emerge from a rather subtle differentiation of the same basic control scheme.

In conclusion, the decomposition into time-varying muscle synergies of the activation waveforms of a large set of shoulder and elbow muscles underlying unconstrained and naturalistic hand movements for catching fast flying balls reveals an intermittent control strategy based on two phases associated to specific synergies and timed according to two key events, ball launch and ball impact. Such strategy may allow for a fast and efficient selection of the appropriate motor commands by incorporating a priori knowledge of the ball flight dynamics in a low-dimensional mapping of the kinematic features of the ball trajectory, extracted from vision, into synergy amplitude and timing recruitment parameters.

### Conflict of interest statement

The authors declare that the research was conducted in the absence of any commercial or financial relationships that could be construed as a potential conflict of interest.
